# When plants produce not enough or at all: metabolic engineering of flavonoids in microbial hosts

**DOI:** 10.3389/fpls.2015.00007

**Published:** 2015-01-29

**Authors:** Emmanouil A. Trantas, Mattheos A. G. Koffas, Peng Xu, Filippos Ververidis

**Affiliations:** ^1^Plant Biochemistry and Biotechnology Laboratory, Department of Agriculture, School of Agriculture and Food Technology, Technological and Educational Institute of CreteHeraklion, Greece; ^2^Department of Chemical and Biological Engineering, Center for Biotechnology and Interdisciplinary Studies, Rensselaer Polytechnic InstituteTroy, NY, USA; ^3^Department of Chemical Engineering, Massachusetts Institute of Technology CambridgeMA, USA

**Keywords:** flavonoid biosynthesis, unnatural flavonoids, metabolic engineering, dynamic regulation, metabolic control, secondary metabolites, combinatorial biosynthesis

## Abstract

As a result of the discovery that flavonoids are directly or indirectly connected to health, flavonoid metabolism and its fascinating molecules that are natural products in plants, have attracted the attention of both the industry and researchers involved in plant science, nutrition, bio/chemistry, chemical bioengineering, pharmacy, medicine, etc. Subsequently, in the past few years, flavonoids became a top story in the pharmaceutical industry, which is continually seeking novel ways to produce safe and efficient drugs. Microbial cell cultures can act as workhorse bio-factories by offering their metabolic machinery for the purpose of optimizing the conditions and increasing the productivity of a selective flavonoid. Furthermore, metabolic engineering methodology is used to reinforce what nature does best by correcting the inadequacies and dead-ends of a metabolic pathway. Combinatorial biosynthesis techniques led to the discovery of novel ways of producing natural and even unnatural plant flavonoids, while, in addition, metabolic engineering provided the industry with the opportunity to invest in synthetic biology in order to overcome the currently existing restricted diversification and productivity issues in synthetic chemistry protocols. In this review, is presented an update on the rationalized approaches to the production of natural or unnatural flavonoids through biotechnology, analyzing the significance of combinatorial biosynthesis of agricultural/pharmaceutical compounds produced in heterologous organisms. Also mentioned are strategies and achievements that have so far thrived in the area of synthetic biology, with an emphasis on metabolic engineering targeting the cellular optimization of microorganisms and plants that produce flavonoids, while stressing the advances in flux dynamic control and optimization. Finally, the involvement of the rapidly increasing numbers of assembled genomes that contribute to the gene- or pathway-mining in order to identify the gene(s) responsible for producing species-specific secondary metabolites is also considered herein.

## Introduction

**Figure 1:**
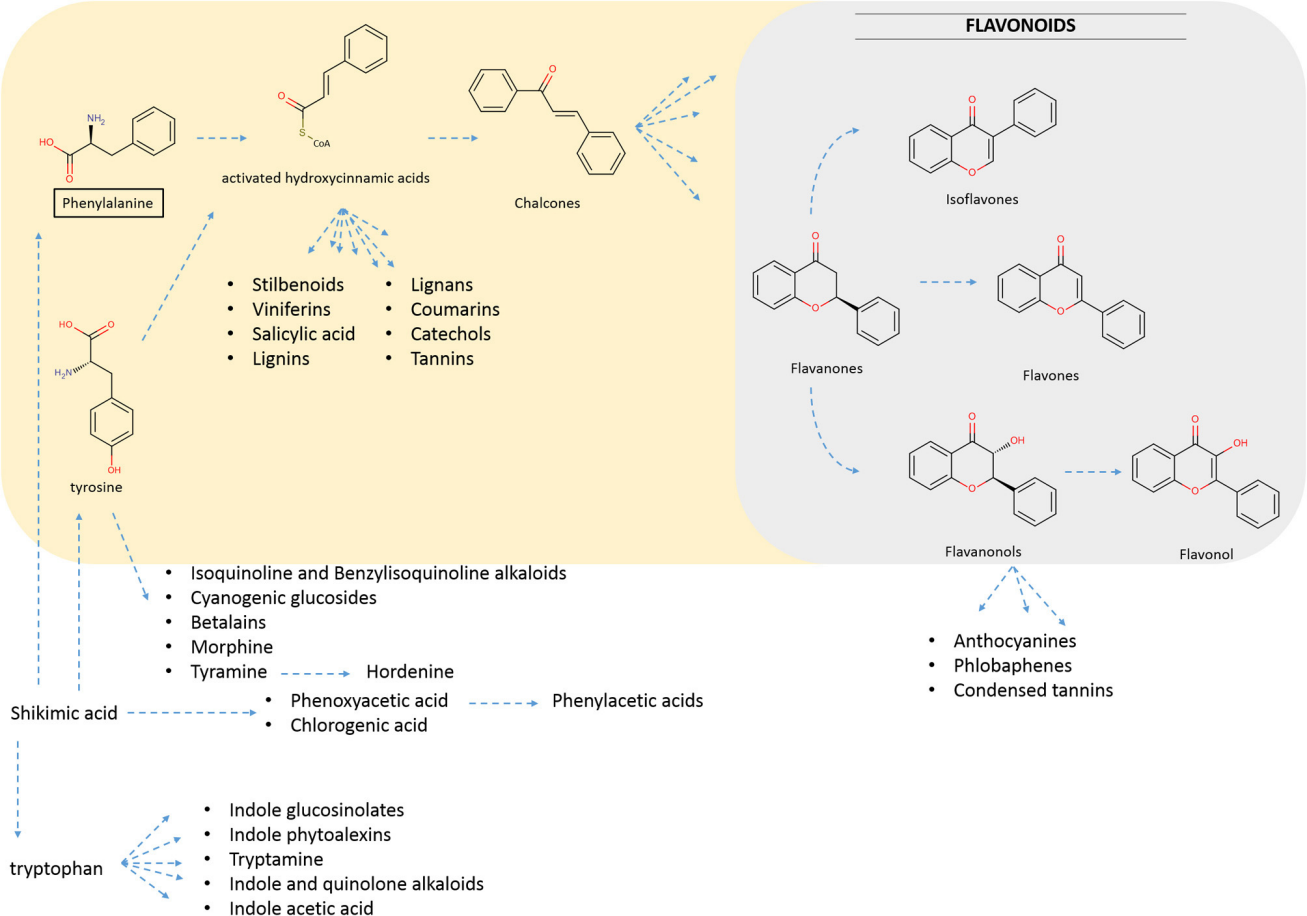
**Brief overview of biosynthetic inter-relations between plant metabolites stemming from Phenylaline (Phe) and Tyrosine (Tyr), which are derived from shikimic acid (Bennett and Wallsgrove, 1994)**. Phe and Tyr act as precursors to the highlighted parts of secondary metabolism (colored). Emphasis is given to phenylpropanoid metabolism (pale yellow colored) leading to flavonoids biosynthesis (pale blue colored).

**Figure 2:**
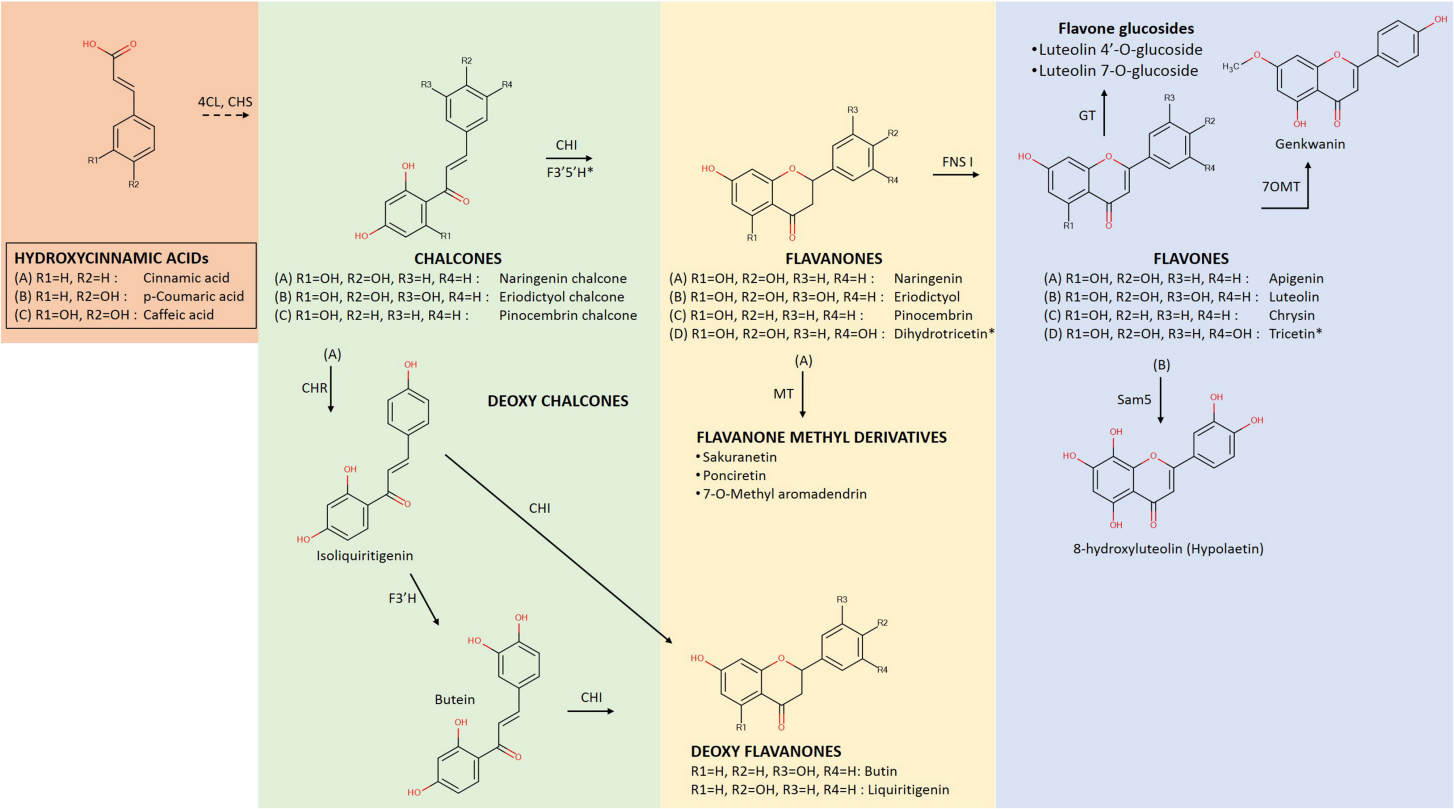
**Early biosynthetic steps of flavonoid metabolism leading to the formation of flavonoid groups of Flavanones and Flavones**. At the initial steps, the flavonoid core molecule is produced from the condensation reaction of activated hydroxinnamic acids with 3 molecules of Malonyl-CoA (not shown) by the action of chalcone synthase (CHS) and subsequent isomeration with the enzyme chalcone isomerase (CHI) for the generation of the flavanone group (Ververidis et al., 2007). Following the action of flavone synthase I (FNS I) the group of flavones is produced. 4CL, hydroxycinnamic acid:CoA ligase; CHR, chalcone reductase; F3′H, flavonoid 3′ hydroxylase; F3′5′H, flavonoid 3′5′ hydroxylase; MT, methyltransferase; GT, glucoside transferase and Sam5: microbial C3H (Lee et al., 2014). Flavonoids indicated with the same lettering (A–D) declare components of certain biosynthetic path. (^*^) Dihydrotricetin is produced from naringenin chalcone (A) by the action of both F3′5′H before the action of CHI, thus it is introduced instead of naringenin into pathway (D) (Andersen and Markham, 2006).

Flavonoids are known to be produced by all terrestrial plants. They comprise a large group of natural compounds deriving from the phenylpropanoid metabolism, which has evolved in plants to produce a large number of inter-related flavonoid structures (Figure 1). Various reviews have appeared that describe in detail the metabolic routes as well as the major and minor groups that flavonoids fall into (Winkel-Shirley, 2001; Andersen and Markham, 2006; Ververidis et al., 2007; Gholami et al., 2014). In the phenylpropanoid metabolism, metabolites branch out to form specific groups like those presented in Figure 1 (i.e., flavonoids, stilbenoids, lignins, etc.). The name “phenylpropanoid” originates from the aromatic phenyl group and the three-carbon tail of the starting phenylalanine, which is then bioconverted first to cinnamic acid, and then to p-coumaric acid (Figure 2). The addition of a coenzyme A group to those hydroxycinnamic acids activates those molecules for the subsequent enzymatic decarboxylation and condensation with three activated malonyl-CoA molecules. This reaction is catalyzed by a polyketide III synthase (Chalcone Synthase, CHS) to create chalcones, the actual precursor molecules of the flavonoid backbone. These are then converted to flavanones through the use of a Chalcone Isomerase (CHI) (Andersen and Markham, 2006). In subsequent steps, diversification is generated by the sequential action of “decorating” enzymes on the flavonoid backbone.

Flavonoids seemed to function as internal regulatory agents (Stafford, 1991), while in later evolutionary stages, they functioned as filters against ultraviolet irradiation (Stapleton and Walbot, 1994). Such flavonoid producing organisms, with either lower or higher cellular complexity, evolved to survive in hostile environments, in terms of both biotic and abiotic stress (Demain and Fang, 2000). Moreover, under the pressure of natural selection, those metabolites, apart from their crucial role in other physiological functions, also constitute important parts of the protection mechanisms, either by direct mortification or by the inhibition of a certain function of the invading organism (Bennett and Wallsgrove, 1994). Such natural plant products that derive from the chorismate and shikimate pathway of primary metabolism, exhibit an impressive chemical diversity caused by plant evolution (Demain and Fang, 2000). Utilizing the molecular mechanisms of their synthesis and their beneficial properties for human health (Ververidis et al., 2007), we will be able to design and create biotechnological tools that will further establish our position in the fields of agriculture and nutrition as well as the pharmaceutical industry, concomitantly promoting consumer health and protection.

However, the extraction of these molecules from plant sources does not result in quantities large enough to meet the increasing market demands. The isolation or purification of natural products using conventional extraction processes, especially regarding those intended for commercial use, appears to be limited due to the low concentration levels of these phytochemicals in plants, as opposed to high plant biomass. Furthermore, these processes are time-consuming, expensive, wasteful in regard to natural resources and, sometimes, environmentally unsafe due to the usage of solvents during the isolation and purification procedures. It has become evident to society, thus attracting the attention of scientists, that industrial needs demand alternative extraction platforms, a significant reduction of costs related to isolation and purification practices and a substantial increase of availability levels.

Recent advances in microbial biotechnology have significantly supported the expression of partial or entire metabolic pathways, allowing the biosynthesis of high value end-products. In this project, we review the current metabolic engineering approaches to achieving exploitable concentrations of natural flavonoid compounds in microorganisms, with potential application in the industry. We also introduce several research efforts that aim to produce unnatural flavonoid analogs (non-natively synthesized by plants) using *in vivo* approaches.

## Diversity of flavonoids as secondary metabolites of agricultural and/or pharmaceutical importance

Several characteristics distinguish flavonoids and other secondary metabolites from substances of the primary metabolism. More specifically: (i) there is a tendency for accumulation in certain tissues or organs, an example being the flavonols in grape skins (Mu et al., 2014). (ii) Their distribution is limited to certain taxonomic units, as evidenced by the isoflavonoid biosynthesis in species of the *Fabaceae* plant family (Reynaud et al., 2005). (iii) They are developmentally regulated, as made obvious by tissue cultures that are unable to produce secondary metabolites, even though the plant cells possess the necessary genetic information (Wink, 2003). Finally, (iv) they are likely to possess biological activity e.g., in organism-organism interactions or in organismal differentiation (Haslam, 1995).

Advancements in metabolomics provided the opportunity to identify accurately the diverse array of chemicals produced by organisms. Such diversity, which is the result of the ongoing evolutionary processes, exists not only between different species or genera but also within the same individual, though not to the same extent. Structural and molecular biology advances have revealed that mutation and gene duplication are the processes responsible for the continuous modifications of the enzymes involved in the flavonoid biosynthesis (Noel et al., 2005). Such mechanisms can result in the production of a wide variety of compounds, due to the action of enzymes and enzyme complexes on the basic structure of metabolic pathways. The enzymes involved in flavonoid biosynthetic pathways appear to have evolved in this way, thus causing catalysis that can lead either to region-specific condensation or to glycosylation, acylation, prenylation, sulfation, methylation or isomerization. In that respect, it appears that a flavonoid-producing organism is able to synthesize a core molecule, such as the flavanone naringenin. This molecule is then likely processed downstream by several classes of enzymes (e.g., hydroxylases, isomerases, etc.,) in order to form the flavonoid end-products, such as eriodictyol or dihydroxykaempferol [for pathways see Figure 2 of this review and Figure 3 in Ververidis et al. (2007)]. Moreover, it is a common feature of many organisms to use enzymes that can utilize their activity on different kinds of substrates. For example, the enzyme flavonol synthase catalyzes the oxidation of flavanonol to flavonol using either dihydrokaempferol or dihydroquercetin as a substrate, and producing kaempferol or quercetin respectively. Such mixtures of biosynthesized metabolites result in the formation of chemical shields used either to defend or to adapt (Dixon and Paiva, 1995; Harborne and Williams, 2000).

The question arising is why there is such a chemical diversity. In an attempt to answer this, we will shed light on several principles of evolutionary biology. Natural selection is the only explanation for adaptation and it can act on populations only if there is variation among its members and if such a variation is random with respect to the direction of adaptation (Jenke-Kodama et al., 2008). By definition, the extent of genetic diversity of a population is proportional to the potential of the same population to adapt to environmental changes. However, by purposely extending the meaning of genetic diversity, it can be considered equal to chemical diversity. According to this principle, populations characterized by significant genetic heterogeneity are consequently also characterized by chemical heterogeneity. This heterogeneity may be the result of the existence of a large number of gene alleles that perform the same core chemical reaction (e.g., C-3 hydroxylation of flavanones). Following this, different enzyme variants may accept different (similar) substrates and perform the same chemical reaction for the production of different end-products. These individuals are more likely to be preserved in a changing environment, since a rich genetic stock is more likely to contain genes that favor the adaptation of individuals in the new environmental conditions. This idea also agrees with Firn's assumption, which states that it is a rare property for any molecule to possess a potent biological activity. Thus, organisms have to generate as much chemical variability as possible in order to increase the probability to find a molecule that fits into a certain function (Firn and Jones, 2000).

This also provides the potential to produce many different structures by minimizing genetic resources. If, for example, a set of seven genes is needed for the production of kaempferol from phenylalanine, an evolutionary acquired allelic variation that hydroxylates the C-3′ position of the B flavonoid ring (i.e., flavonoid 3′hydroxylase; F3′H), will lead the flavonol synthase (FLS) to the production of two flavonols instead of one; kaempferol and quercetin (Trantas et al., 2009). Similarly, the expression of a resveratrol synthase (RS) in grapes that has arisen from a chalcone synthase (CHS) duplication (Rausher, 2006), is responsible for the production of resveratrol that belongs to a new class of non-flavonoid phenylpropanoid compounds. Therefore, it appears that RS and CHS are responsible for the diversification of phenylpropanoid metabolism leading to the production of stilbenoids, flavonoids and their descendants respectively (Ververidis et al., 2007).

Given the large number of organisms and thus the large number of existing interactions, the number of secondary metabolites taking part in the adjustment mechanisms is expected to be extremely high. It is therefore evident that nature is a unique source of input in the effort to find and create new potent medicinal products.

## Flavonoid combinatorial biosynthesis of agricultural/pharmaceutical importance

Over the past few decades, the inadequate availability of source materials from which phytochemicals are extracted, as well as the complexity of the production of these products through conventional chemical synthesis, resulted in the gradual reduction of the Pharma- Food- and Agro- industry's interest in developing natural product-based commercial applications. It is often commercially unfeasible to synthesize chemically bioactive molecules, originally produced from plants with some structural complexity combining low cost production and demanding purity. Even though the chemical synthesis allows targeted design of novel compounds with improved or even new functions, the metabolic engineering techniques appears to be gaining grounds (Otero and Nielsen, 2010). Since natural plant products usually have no nutritional value but when included in a diet can boost the immune system or protect the human body from free radicals or even prevent or suppress carcinogenesis, marketed demand will continue to peak. Moreover, due to the limited chemical diversity and structural complexity of chemical synthetic libraries, as well as due to the great success of natural product-derived drugs on the market in the past few years, screening of untapped biological resources for new natural products is expected to be continued in the future (Marienhagen and Bott, 2013).

Many biosynthetic pathways for natural plant products have been elucidated through the advancements in DNA sequencing, in combination with new recombinant DNA technologies. Furthermore, innovative technologies have emerged relying on bacterial, yeast or plant cell-based biotransformations targeting *in vivo* biosynthesis for the large-scale production of natural products under controlled conditions, in an attempt to generate novel natural products, and for the production of rare and expensive natural products (Mora-Pale et al., 2014). Such production strategies are highly specific, fully controlled and do not contribute to environmental pollution. What is more, the recovery of the resulting products is considerably easier than natural product extraction or conventional chemical synthesis, since fewer side products and less waste is generated. To this end, industrial biotechnology flourished. Significant work has been done to generate a broad number of natural products, their analogs, as well as different classes of useful intermediates such as isoprenoids, flavonoids, stilbenes, polysaccharides and glycoproteins, and alcohols (Abdullah et al., 2008). These substances have potential applications as pharmaceuticals, fine chemicals and biofuels (Otero and Nielsen, 2010; van Summeren-Wesenhagen and Marienhagen, 2013).

Since the mid-1980's, advances in genetic engineering methodology led to the development of an alternative approach, described as combinatorial biosynthesis, to generate new molecules from natural products. The reason for this is that plants constitute an extremely rich source of bioactive products that possess a huge potential for drug discovery. Combinatorial biosynthesis comprises a series of methods that establish novel enzyme-substrate combinations *in vivo* that lead to the biosynthesis of new, natural or even unnatural compounds, which can be used in drug discovery programs (Chemler et al., 2007). The techniques involved in combinatorial biosynthesis can be summarized into the following categories (Pollier et al., 2011; Cress et al., 2013): (i) bio-transformation, in which natural or non-natural compounds are modified by different types of biocatalysts (whole cells, cell extracts, or purified enzymes); (ii) feeding of mutated (recombinant) biocatalysts with non-natural precursor compounds, the latter leading to mutasynthesis, a semi-synthetic methodology (Chemler et al., 2007; Bhan et al., 2014); (iii) combinatorial metabolism in hybrids that evolved from crossings within different accessions, cultivars, or genotypes of a single plant species, or even interspecific sexual or somatic hybridization; (iv) activation of silent metabolism through the activation of relevant transcription factors; and (v) synthetic biology or the combinatorial compilation of genes in heterologous (plant) hosts.

In this section, we discuss some examples of state-of-the-art combinatorial biosynthesis methods that have generated flavonoid molecules from microbial hosts. In that respect, an important application of metabolic engineering is the production of diversified natural compounds. A combinatorial biosynthesis strategy was developed by Naesby and his coworkers who employed modified eYACs (expressible Yeast Artificial Chromosomes) for the expression of plant genes in yeast (*Saccharomyces cerevisiae*) (Naesby et al., 2009). The use of these vectors allowed the combined expression of a large number of genes from various sources intending to function in random combinations leading to the biosynthesis of various flavonols. This combinatorial approach resulted in the creation of 5–7 step variable pathway assemblies, each converting the phenylalanine and/or tyrosine yeast metabolites into flavonoids, normally only produced by plants. When randomly picked clones were analyzed, approximately half of them showcased a production of the flavanone naringenin, and a third of them produced the flavonol kaempferol in various amounts.

Combinatorial biosynthesis is a method that establishes novel enzyme-substrate combinations *in vivo*. Innovative strategies that combine enzymes that apparently do not function together in nature, can direct transgenic hosts to the heterologous production of rare natural products that do not accumulate *in plants* (Fukushima et al., 2013). Consequently, the application of synthetic biology methodologies may result in the development of new drugs through the combinatorial biosynthesis of plant-derived natural products (Cress et al., 2013; Gholami et al., 2014). Such potential has been demonstrated by the production of diverse flavonoid compounds from microbes when fed with various natural and unnatural (e.g., halogenated) cinnamic acid derivatives, that are potentially important to the pharmaceutical industry (Chemler et al., 2007).

Another reason why flavonoids have become interesting is because they have been proven to act as microbial deterrents and anti-infection agents in plants (Trantas et al., 2009). Fowler and his coworkers, while working trying to increase the flavonoid effectiveness against some infections by enabling the transport of a toxic molecule into the infecting species, demonstrated that combinatorial synthesis of non-natural flavanones could identify novel anti-microbial agents with activity against bacteria and fungi, but with minimal toxicity to human cells (Fowler et al., 2011). Moreover, it has been demonstrated that by means of combinatorial mutasynthesis flavonoids analogs from acrylic acids can be produced in large and highly pure quantities using recombinant production platforms (Chemler et al., 2007). In that work, Chemler and his coworkers were able to biosynthesize novel flavanones and dihydroflavonols from a number of aromatic acrylic acids. This once again indicated that the flavonoid network exhibits broad substrate specificity. Mutasynthesis involves the chemical synthesis of non-natural substrates that are similar in structure to natural substrates. After a library of non-natural analogs is created, enzymatic conversion of the non-natural analogs is performed by mutated (recombinant) biocatalysts to isolate novel non-natural compounds. The results can then be assessed to elucidate mechanisms of enzymatic catalysis and to determine substrate specificity requirements (Bhan et al., 2014). This so-called semi-synthetic approach, or the combination of chemical synthesis and biosynthesis, has also been utilized for the production of non-natural isoflavonoids. These phytochemical derivatives have the potential to be utilized in human therapeutics, as the microbes catalyzing these novel reactions have been isolated from the human gut and are supposed to have beneficial health impacts on their human hosts (Cress et al., 2013).

## A current update on metabolic engineering data applied to pharmacologically and industrially important flavonoids—emphasis on novel and unnatural bioactive metabolites

In the previous section, it has been mentioned that combinatorial biosynthesis has been used to generate various natural or unnatural forms of flavonoids in various microbial hosts. Plants that can also be used as hosts have been assessed as less advantageous compared to microbial systems, due to the existence of technical issues associated with them (Mora-Pale et al., 2013). Biosynthesis of natural products in microbial hosts or in plants uses energy sources derived from primary metabolism. Shikimate metabolism feeds precursor amino acids to the phenylpropanoid pathway (Figure 1). Successful flux diversion from the primary metabolism to heterologous secondary pathways depends on many factors (host strain, nutrition media, growth conditions etc.).

Two main approaches are applied for the production of flavonoids in heterologous hosts. In the precursor supplementing approach, the heterologous pathway is fed with either phenylalanine or tyrosine that is then converted into the corresponding flavonoid (Figure 1). Although supplementing a microbial culture with an expensive precursor might be feasible for a small-scale experiment, it severely impedes industrial applicability. Renewable, simple and cheap carbon sources, such as glucose and glycerol, are especially desired for the production of pharmaceutical or any other industrial chemical or protein.

In the second approach, the engineer seeks to produce a fermentation product with low needs in energy input. To achieve this, genetic modification of the host strain is needed in order for it to become an aromatic amino acid overproducer, thus resolving the issue of its low initial precursor availability. During a fermentation process, the available aromatic amino acids intended for the flavonoid pathway and provided by the primary metabolism, may not be enough to feed substantially the heterologous pathway. The utilization of aromatic amino acid overproducers would demonstrate optimal economic performance regarding the fermentation outcome of the targeted flavonoid biosynthesis.

Currently, the most utilized biological platforms metabolically engineered for the heterologous production of flavonoids are the microbial biofactories *Escherichia coli* and the budding yeast *S. cerevisiae*. The strongest argument for utilizing microorganisms for metabolic engineering of natural plant products is the high degree of their genetic tractability (Cress et al., 2013). Moreover, numerous data sets and molecular biology tools are available for facile genetic manipulation, characterization, modeling, and scale-up. This genetic flexibility reduces experimental inadequacies thus limiting unknown factors, and allows for faster, more predictable experimentation and data collection. To functionally express a set of genes for the reconstitution of a heterologous pathway, a researcher has to deal with a series of parameters e.g., host selection, gene source selection, expression system, promoter strength, plasmid/gene copy number, aeration, temperature, pH, or nutrient supplementation. Optimization of those parameters will result in improved fermentation conditions so that the carbon flow will be optimal (Mora-Pale et al., 2013), resulting in increased yields of the target compound at the given set of parameters employed.

This multi-factorial approach gives space for various combinations that will eventually result in a wide range of final titers. Data from independent experiments for the biosynthesis of the flavanone pinocembrin showed final flavanone titers ranging from 40 to 429 mg/L (Table 1). In Table 1, reported data concerning the heterologous production levels from various flavonoid groups can be found. Particular effort is made to compare these titers with the levels of those plant produced flavonoids. As discussed in the previous section, combinatorial biosynthesis protocols have recently provided us with the opportunity to produce unnatural flavonoid molecules (not produced from plants or any other organism).

**Table 1:** **Levels of natural flavonoid products produced either during heterologous biosynthesis in various hosts (*Escherichia coli* or *Sacharomyces cerevisiae*), or as estimated concentrations from plant sources, (values for plant sources were retrieved from phenol-explorer database Rothwell et al., 2013; Bhagwat et al., 2014)**.

**Flavonoid target**	**Plant source**		**Metabolically Engineered host**	**Externally fed precursor**	**Titer (mg/L)**	**References**
	**Common name (*Systematic name*)**	**mg/100 g FW**				
**FLAVANONES**						
Pinocembrin	Mexican oregano (*Lippia graveolens*)	499.3	*E. coli*	Glucose	40	Wu et al., 2013
			*E. coli*	Cinnamic acid	429	Leonard et al., 2007
Eriodictyol	Mexican oregano (*Lippia graveolens*)	85.33	*E. coli*	Tyrosine	107	Zhu et al., 2014
Naringenin	Mexican oregano (*Lippia graveolens*)	372	*S. cerevisiae*	phenylalanine	8.9	Trantas et al., 2009
	Grapefruit (*Citrus x paradisi*)	53				
	*Tomato* (*Solanum lycopersicum*)	3.84	*S. cerevisiae*	Glucose	108.9	Koopman et al., 2012
Sakuranetin	Mexican oregano (*Lippia graveolens*)	93	*E. coli*	Glucose	42.5	Kim et al., 2013b
Ponciretin (isosakuranetin)	–	–	*E. coli*	Glucose	40.1	Kim et al., 2013b
7-O-Methyl aromadendrin	–	–	*E. coli*	p-coumaric acid	2.7	Malla et al., 2012
**FLAVONES**						
Apigenin	*Petroselinum crispum*	302	*E. coli*	Tyrosine	13	Miyahisa et al., 2006
	Celery (*Apium graveolens*)	56.25				
Apigenin glucosides	Mexican oregano (*Lippia graveolens*)	28.33	*E. coli*	Apigenin	4.67	Choi et al., 2012
	*Olea europaea*	8.2				
Chrysin	–	–	*E. coli*	Phenylalanine	9.4	Miyahisa et al., 2006
Chrysin glucosides	–	–	*E. coli*	Chrysin	Not measurable	Choi et al., 2012
Hypolaetin (8-hydroxyluteolin)	–	–	*E. coli*	Luteolin	88	Lee et al., 2014
Luteolin 4′-O-glucosides	–	–	*E. coli*	Luteolin	10.86	He et al., 2008
Luteolin 7-O-glucosides	Mexican oregano (*Lippia graveolens*)	*297.67*	*E. coli*	Luteolin	6.52	He et al., 2008
	Olive (*Olea europaea*)	*14.5*				
**FLAVONOLS**						
Kaempferol	(Capers) *Capparis spinosa*	*104.29*	*E. coli*	Dihydrokaempferol	n.e.	Xu et al., 2012
			*E. coli*	Naringenin	n.e.	Xu et al., 2012
			*E. coli*	Naringenin	n.e.	Lukacin et al., 2003
	Anethum (*Anethum graveolens*)	*26.7*	*E. coli*	p-Coumaric acid	0.3	Leonard et al., 2006
			*S. cerevisiae*	Phenylalanine	1.3	Trantas et al., 2009
			*E. coli*	kaempferol	28.6	Kim et al., 2006
			*E. coli*	Phenylalanine	15.1	Miyahisa et al., 2006
Kaempferol 3-O-glucoside (Astragalin)	Bean (*Phaseolus vulgaris*)	*39.88*	*E. coli*	Kaempferol	13.56	(He et al., 2008)
			*E. coli*	Naringenin	109.3	Malla et al., 2013
Quercetin	(Capers) *Capparis spinosa*	*32.82*	*E. coli*	p-Coumaric acid	0.05	Leonard et al., 2006
			*S. cerevisiae*	Phenylalanine	Traces	Trantas et al., 2009
			*E. coli*	Quercetin	30.2	Kim et al., 2006
Quercetin 3-O-glucoside	Bean (*Phaseolus vulgaris*)	*6 × 10^−3^*	*E. coli*	Quercetin	11.54	He et al., 2008
3-O-Xylosyl quercetin	–	–	*E. coli*	Quercetin	23.78	Pandey et al., 2013
Kaempferol-3-O-rhamnoside	Endive (*Cichorium endivia*)	*0.16*	*E. coli*	Kaempferol	150	Kim et al., 2012b
Quercetin-3-O-rhamnoside	Lingonberry (*Vaccinium vitis-idaea*)	*12.2*	*E. coli*	Quercetin	200	Kim et al., 2012b
	Olive (*Olea europaea*)	*4.1*				
**ISOFLAVONONES**						
Genistein	*Soy* (*Glycine max*)	*30.76*	*E. coli*	Naringenin	16.2	Kim et al., 2009
			*S. cerevisiae*	Naringenin	n.e.	Akashi et al., 1999
			*E. coli*—*S. cerevisiae* Co-cultivation	Tyrosine	6	Katsuyama et al., 2007b
			*E. coli*	Naringenin	10 mg/g	Leonard and Koffas, 2007
			*S. cerevisiae*	Phenylalanine	0.1	Trantas et al., 2009
Genistein glucosides	–	–	*E. coli*	Genistein	37.29	Pandey et al., 2014
Genistin	*Soy* (*Glycine max*)	*2.22*	*S. cerevisiae*	Genistein	n.e.	Li et al., 2014
Daidzein	*Soy* (*Glycine max*)	*13*	*E. coli*	Liquiritigenin	18 mg/g	Leonard and Koffas, 2007
3′-Hydroxydaidzein	–	–	*E. coli*	Daidzein	75	Lee et al., 2014
Daidzin	*Soy* (*Glycine max*)	*1.27*	*S. cerevisiae*	Daidzein	n.e.	Li et al., 2014
Ononin	–	–	*S. cerevisiae*	Formononetin	n.e.	Li et al., 2014
Sophoricoside	–	–	*E. coli*	Genistein	n.e.	Ruby Santosh Kumar et al., 2014

**Figure 3:**
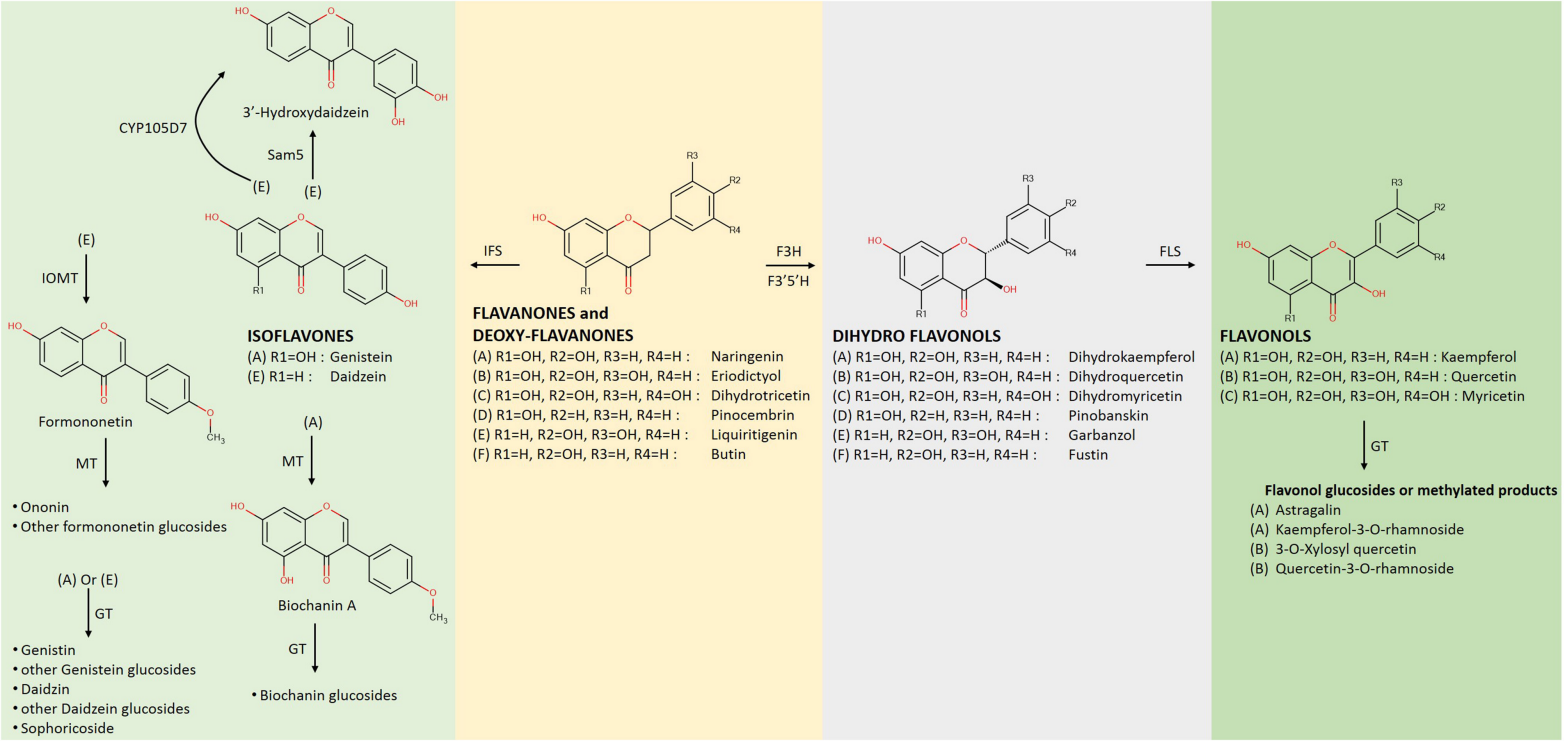
**Late biosynthetic steps of flavonoid metabolism downstream of flavanones (Continuation of Figure 2); Dihydroflavonols are created by a flavanone 3β–hydroxylase (F3H) acting on flavanones that are transformed into flavonols by the action of a flavonol synthase (FLS) (Ververidis et al., 2007)**. The group of isoflavones is created by the action of an isoflavone synthase (IFS) on flavanones. MT, methyltransferase; GT, glucoside transferase; Sam5, microbial C3H (Lee et al., 2014); CYP105D7, flavonoid 3′ hydroxylase; F3′H, flavonoid 3′ hydroxylase; F3′5′H, flavonoid 3′ and 5′ hydroxylase; IOMT, isoflavone 4′-O-methyl transferase.

*For structure details and metabolic steps see Figures 2, 3*.

### Flavanones

Flavanones represent a group of compounds with very interesting pharmacological properties (Ververidis et al., 2007). They are the first synthesized flavonoid compounds, from which all other flavonoids are generated (Figure 2). There are many examples of constructed microbial strains that accept a relevant precursor molecule and bio-convert it into the corresponding flavanone. For example p-cinnamic acid, phenylalanine, and tyrosine were converted into pinocembrin, naringenin and eriodictyol at titers 429 mg/L (*E. coli*, Leonard et al., 2007), 8.9 mg/L (*S. cerevisiae*, Trantas et al., 2009), and 107 mg/L (*E. coli*, Zhu et al., 2014) respectively, (Table 1). In all above cases, the expression of the implicated genes was induced and resulted in different but relatively low titers. However, Leonard et al. (2007) as well as Zhu et al. (2014), engineered their heterologous hosts by boosting primary metabolism to produce increased levels of malonyl-CoA. In a similar set-up, *E. coli* was engineered to provide a pool of malonyl-CoA needed to produce 7-O-methyl aromadendrin from p-coumaric acid at a concentration of 2.7 mg/L (Malla et al., 2012). Extending their strategy, Zhu et al. (2014) further utilized a truncated version of F3′H which was fused with a CPR. This was necessary as P450 classified F3′Hs require to be coupled with an electron donor cytochrome P450 reductase (CPR), since bacteria lack endogenous transport systems to support the full catalytic activity of P450 enzymes (Sevrioukova et al., 1999).

However, the most interesting cases were the ones where no precursor amino acid was supplemented and the host systems were able to utilize glucose energy for the production of pinocembrin and naringenin, at concentrations of 40 mg/L (Wu et al., 2013) and 108.9 mg/L (Koopman et al., 2012), respectively (Table 1). In the first case, Wu and his co-workers evaluated the use of gene encoding for feedback-insensitive enzymes to increase the pool of intra-cellular phenylalanine. In the latter case, Koopman and his co-workers used mutated *S. cerevisiae* strains, which hindered the metabolite flow toward minor branches of primary metabolism. More complex flavanones like sakuranetin or ponciretin were produced at 42.5 or 40.1 mg/L, respectively by *E. coli* strains, when the core naringenin pathway was linked to an O-methyltransferase (Figure 2) from rice or soybean (Kim et al., 2013b).

### Flavones and flavonols

Flavones and flavonols constitute two different groups of flavonoids. However, they are both generated by equivalent (C2–C3) desaturation reactions in the flavonoid γ-pyrone in the C flavonoid ring of a flavanone or flavanonol, respectively. Enzymes FNS I (Figure 2) and FLS (Figure 3) catalyze the oxidation of the respective substrates by introducing a C2–C3 double bond (Martens et al., 2003). Flavanonoles (also termed dihydroflavonols) are the flavonoid products deriving from the hydroxylation of flavanones at the C3 position (Figure 3). The flavone aglycones apigenin and chrysin have been produced in *E. coli* at 13 and 9.4 mg/L, respectively (Miyahisa et al., 2006). However, only apigenin glucoside was measured at 4.67 mg/L (Choi et al., 2012) (Table 1). Recently, hypolaetin, another flavone, was produced from luteolin at 88 mg/L, with the use of a heterologous monooxygenase cloned from the bacterium *Saccharothrix espanaensis* (Lee et al., 2014).

Flavonols constitute one of the most attractive groups of flavonoids. They are two biochemical steps away from the flavanones (Figure 3). Regarding their biosynthesis, a hydroxylating enzyme (Lee et al., 2014) acts on a flavanone before the flavonol synthase acts on the generated dihydroflavonol (Holton et al., 1993). Furthermore, types of FLS enzymes that possess interesting bi-functional activity have been cloned from *Ginkgo biloba* (Xu et al., 2012) and *Citrus unshiu* (Lukacin et al., 2003), which aside from their conventional activity may act on the flavanone naringenin and convert it directly into kaempferol as demonstrated in the case of *E. coli*. Following steps similar to the ones described above, various forms of flavonols have been generated from exogenously supplemented precursor molecules. Kaempferol and quercetin constitute well-known representatives of this group, both produced in *E. coli* at concentrations of 0.3 and 0.05 mg/L, respectively (Table 1), starting with p-coumaric acid as the precursor (Leonard et al., 2006). When the full pathway for the production of kaempferol from phenylalanine, along with the genes leading to an increase of the internal malonyl-CoA pool were introduced in *E. coli*, 15.1 mg/L of the flavonol were biosynthesized (Miyahisa et al., 2006). When a similar approach was followed in *S. cerevisiae*, an amount of 1.3 mg/L of kaempferol was produced from phenylalanine (Trantas et al., 2009). None of these compounds or any other flavonols have been directly produced from the main feeding carbon source (e.g., glucose) without the exogenous supplementation of any precursor.

Moreover, various forms of flavonols have been generated by the action of various decorating enzymes. A common biotransformation is the attachment of sugars to the hydroxyl groups of the flavonoid backbone through the action of glycosyl-transferases (Figure 3). Following this method, *E. coli* strains were capable of producing astragalin at 109.3 mg/L from naringenin (Malla et al., 2013) or 13.56 mg/L from kaempferol (He et al., 2008) and 23.78 mg/L of 3-O-xylosyl quercetin from quercetin (Pandey et al., 2013), (Table 1). Other similar attempts to obtain glycosyl-transferases from various sources include the biotransformation of apigenin, chrysin, luteolin, kaempferol, and quercetin to their 3-O-, 7-O-, or 4′-O-glucosides (He et al., 2008; Choi et al., 2012). The utilization of a rhamnose flavonol glycosyltransferase on kaempferol and quercetin was adequate for the production of the corresponding 3-O-rhamnosides at concentrations of 150 and 200 mg/L, respectively (Kim et al., 2012b).

### Isoflavonones

Isoflavonoids are mostly known thanks to their interaction with the human and animal estrogen receptors (Cress et al., 2013). It is not a uni-variate class of compounds but rather consists of more than one subclasses (e.g., isoflavones, isoflavanes) which are modified in later steps (Dixon and Steele, 1999); more than 1600 isoflavonoid derivatives have been identified to date (Pandey et al., 2014). As with other classes, *E. coli* and *S. cerevisiae* are the most common hosts for heterologous production of isoflavonoids. The main reaction in the biosynthesis of isoflavonoids is the region-specific movement of the flavonoid B-ring from the C-2 to the C-3 position by the action of the P450 enzyme isoflavone synthase (IFS, Figure 3) (Pandey et al., 2011).

The first attempt to transform a flavanone into the corresponding isoflavanone in microbes was accomplished by Akashi et al. (1999), who expressed an IFS gene from licorice in yeast cells to transform naringenin into genistein. In their work, the requirement for a CPR was covered by endogenous yeast reductases. In an alternative approach, Kim et al. (2009) managed to produce genistein in *E. coli*. In that case, the IFS from red clover was engineered to fuse in-frame with a CPR cloned from rice. The resulted protein could readily convert naringenin into genistein at up to 15.1 mg/L (Table 1). When Leonard et al. (2009) expressed an engineered chimeric IFS in *E. coli, they* enabled the robust production of genistein and daidzein from the supplemented naringenin and liquiritigenin at concentrations of 10 and 18 mg/g, respectively. The engineered IFS was constructed as a fusion of the IFS cloned from *Glycine max* with the CPR cloned from *Catharanthus roseus*, from which the membrane spanning hydrophobic regions were removed and a mammalian leader sequence was added. In a different approach, the implementation of a co-cultivation of *E. coli* and *S. cerevisiae* strategy, where the naringenin produced by *E. coli* was transformed into genistein by *S. cerevisiae* cells carrying an IFS gene, led to the production of up to 6 mg/L of genistein (Katsuyama et al., 2007b).

The conversion rate of a precursor to the final product may be high when referring to mono-enzymatic reactions. However, this does not always apply to cases of multi-enzyme reactions. Trantas et al. (2009) attempted to reconstruct the whole biosynthetic pathway for the biosynthesis of genistein in *S. cerevisiae* by introducing seven genes under GAL promoters. When the production system was supplied with phenylalanine, yeast cells transformed with the full pathway produced 0.1 mg/L of genistein. When the same system was supplied with p-coumaric acid or naringenin, 0.14 or 7.7 mg/L of genistein were produced (Table 1), indicating rate limiting steps or a diversion of the metabolic flux toward genistein from upstream metabolic steps.

Furthermore, glycosylated variants of isoflavonoids have been successfully produced via the utilization of different glycosyl-transferases cloned from various sources, in order to develop new biological activities. As a result, genistein, daidzein, or formononetin have been successfully transformed into daidzin, genistin, or ononinin in *S. cerevisiae* cells through the utilization of a glycosyl-transferase from *Pueraria lobata* (Li et al., 2014) or in *E. coli* through the utilization of a glycosyl-transferase deriving from *Bacillus licheniformis* (Pandey et al., 2014), or from *Glycyrrhiza echinata* (Nagashima et al., 2004) (Figure 3, Table 1). Sophoricoside, a 4′ glucoside of genistein was produced in *E. coli* when a glycosyl-transferase from *Bacopa monniera* was utilized (Ruby et al., 2014). Recently, 3′- hydroxydaidzein was produced at 75 mg/L from daidzein with a heterologous monooxygenase cloned from the bacterium *S. espanaensis* (Lee et al., 2014).

Equol constitutes another interesting example of the isoflavonoids (isoflavan subclass). It exhibits biological properties that exceed those of its precursor daidzein and can be used as pharmaceutical or nutraceutical agent for a number of hormone-dependent disorders due to its resemblance with the human hormone estradiol (Setchell and Clerici, 2010b). While much of the interest in equol is centered around its estrogenic effects, there are many other biological properties with potential to be of value in treating diseases in many clinical areas, including cancer, cardiovascular disease, osteoporosis, as well as menopausal symptoms (Setchell et al., 2002). Another interesting member of the isoflavan group is 5-hydroxy-equol, also exhibiting increased antioxidant activity compared to its precursor genistein (Schroder et al., 2013). Both those isoflavans cannot be biosynthesized in nature but are encountered as daidzein or genistein byproducts after the action of gut micro-flora (Setchell and Clerici, 2010a). Recently, the genes responsible for their metabolism were identified from *Slackia isoflavoniconvertens* and functionally expressed in *E. coli* (Schroder et al., 2013).

### Unnatural flavonoids

Although flavonoids have been studied extensively, so far we have only discussed natural compounds that can be found in nature. However, as mentioned above, host organisms possess the ability to bioconvert unusual molecules they are fed externally, to produce non-native, thus termed, unnatural flavonoids. Even though plants do not produce these compounds at all, they seem very promising for the pharmaceutical industry thanks to their properties. In some cases, interesting unnatural flavonoids can be produced that may exhibit numerous pharmacological properties, examples being the cases of flavonols (Forbes et al., 2014), bichalcones (Gurung et al., 2010) or flavonoidal alkaloids (Nguyen et al., 2012). Such approaches open the way to the development of new strategies that can lead to the biosynthesis of novel unnatural bioactive compounds.

**Figure 4:**
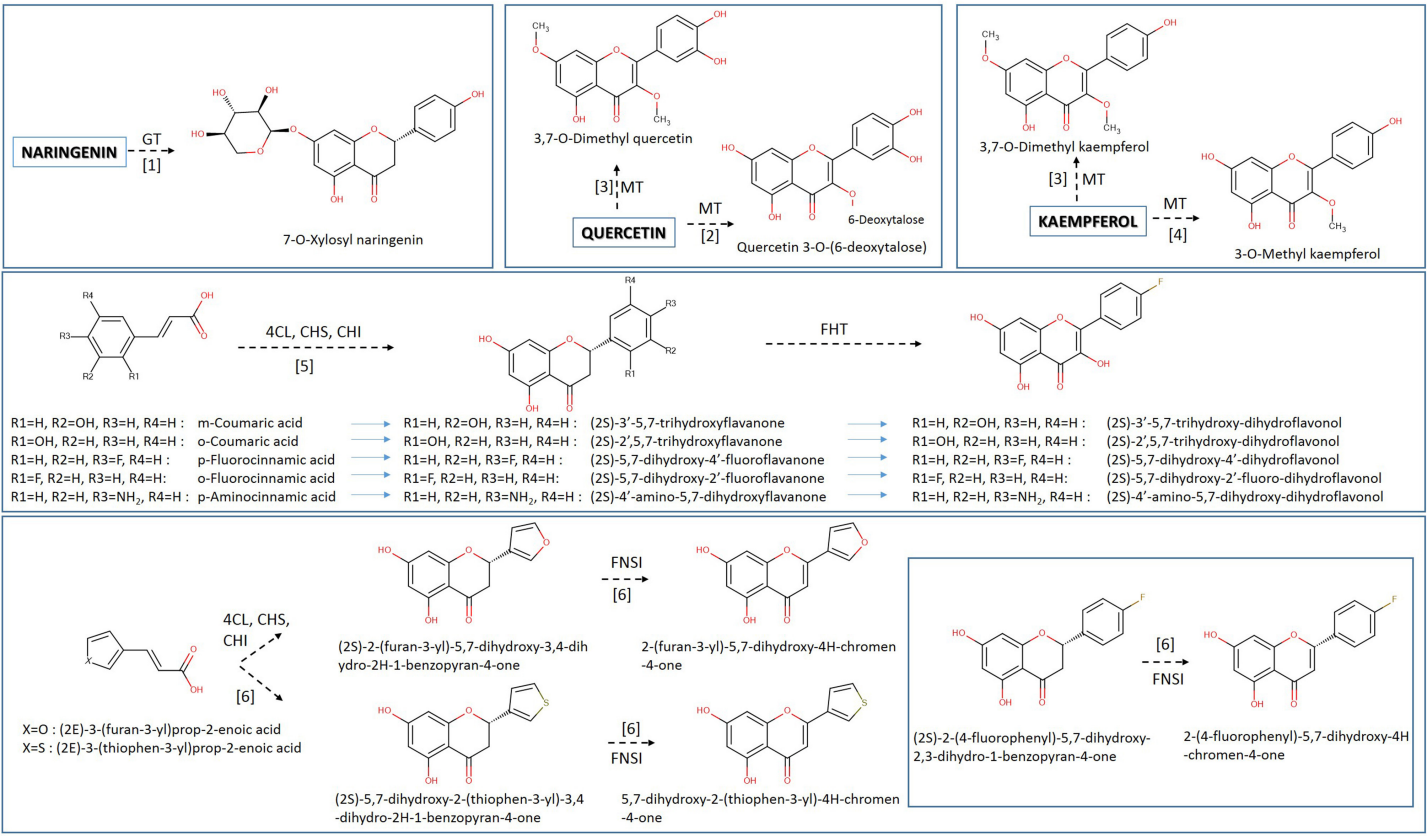
**Heterologous biosynthesis of unnatural flavonoids in *E. coli* host**. Various unnatural hydroxyncinnamic acids and their analogs are utilized for the generation of various unnatural flavanones, flavones, dihydroflavonols, and flavonols. Data were collected from various publications shown by bracketed numbers (1, Simkhada et al., 2009; 2, Yoon et al., 2012; 3, Joe et al., 2010; 4, Kim et al., 2010; 5, Chemler et al., 2007; 6, Katsuyama et al., 2007a). Arrows indicate the course of the biosynthetic pathway. Implicated enzymes: 4CL, hydroxycinnamic acid:CoA ligase; CHS, chalcone synthase; CHI, chalcone isomerase; FNS I, flavone synthase I; MT, methyltransferase; GT, glucoside transferase.

Among the first attempts to produce unnatural flavonoids, Chemler et al. (2007) managed to transform various cinnamic and acrylic acid analogs into the corresponding decorated flavonoids or flavonols (Figure 4) through the use of engineered *S. cerevisiae* strains containing a set of core flavonoid genes. They relied on the broad substrate specificity of the utilized genes that were able to biotransform the supplied precursor molecules through sequential enzymatic activities, producing unnatural flavanones at titers of 2.81 to 15.82 mg/L (Table 2). A similar approach was used by Katsuyama et al. (2007a), who succeeded in producing an array of 36 unnatural flavonoids (16 of which are novel in bibliography) that belong to the classes of flavanones (45–102 mg/L), flavones (26–46 mg/L), and flavonols (0.5–33 mg/L). Katsuyama et al. (2007a) used recombinant *E. coli* bearing selected genes from the phenylpropanoid pathway to direct the construction of appropriate novel natural and unnatural plant analogs of flavonoids and polyketides when fed with various precursor molecules. These analogs of flavones and flavonols were biosynthesized from their respective natural or unnatural carboxylic acids, which served as precursors for the action of post-polyketide modification enzymes, such as flavones synthase I (FNSI) and flavanone 3-hydroxylase/flavonol synthase (F3H/FLS), respectively (Figures 2, 3).

**Table 2:** **Levels of unnatural flavonoid products obtained using metabolically engineered *E. coli* and *Saccharomyces cerevisiae* hosts**.

**Flavonoid target**	**Engineered organism**	**Externally fed precursor**	**Titer (mg/L)**	**References**
**FLAVANONES**				
7-O-Xylosyl naringenin	*E. coli*	Naringenin	n.e.	Simkhada et al., 2009
(2S)-2-(4-fluorophenyl)-5,7-dihydroxy-2,3-dihydro-1-benzopyran-4-one		(2E)-3-(4-fluorophenyl)prop-2-enoic acid	102	Katsuyama et al., 2007a
(2S)-2-(furan-3-yl)-5,7-dihydroxy-3,4-dihydro-2H-1-benzopyran-4-one		(2E)-3-(furan-3-yl)prop-2-enoic acid hydrate	53	Katsuyama et al., 2007a
(2S)-5,7-dihydroxy-2-(thiophen-3-yl)-3,4-dihydro-2H-1-benzopyran-4-one		(2E)-3-(thiophen-3-yl)prop-2-enoic acid hydrate	45	Katsuyama et al., 2007a
(2S)-3′-5,7-trihydroxyflavanone		m-Coumaric acid	6.54	Chemler et al., 2007
(2S)-2′,5,7-trihydroxyflavanone		o-Coumaric acid	6.36	Chemler et al., 2007
(2S)-5,7-dihydroxy-4′-fluoroflavanone		p-Fluorocinnamic acid	2.81	Chemler et al., 2007
(2S)-5,7-dihydroxy-2′-fluoroflavanone		o-Fluorocinnamic acid	6.54	Chemler et al., 2007
(2S)-4′-amino-5,7-dihydroxyflavanone		p-Aminocinnamic acid	15.82	Chemler et al., 2007
**FLAVONOLS**				
Quercetin 3-O-(6-deoxytalose)	*E. coli*	Quercetin	98	Yoon et al., 2012
3-O-Methyl kaempferol		Naringenin	22.5	Kim et al., 2010
3,7-O-Dimethyl quercetin		Quercetin	19.2	Joe et al., 2010
3,7-O-Dimethyl kaempferol		Kaempferol	22	Joe et al., 2010
Quercetin 3-O-N-acetylglucosamine		Quercetin	380	Kim et al., 2012a
Quercetin 3-O-glucoside-7-O-rhamnoside		Quercetin	67	Kim et al., 2013a
3,7-O-bisrhamnoside		Quercetin	67.4	Kim et al., 2013a
2-(4-fluorophenyl)-3,5,7-trihydroxychromen-4-one		(2E)-3-(4-fluorophenyl)prop-2-enoic acid	Trace	Katsuyama et al., 2007a
(2S)-3′-5,7-trihydroxy-dihydroflavonol	*S. cerevisiae*	m-Coumaric acid	2.98	Chemler et al., 2007
(2S)-2′,5,7-trihydroxy-dihydroflavonol		o-Coumaric acid	3.85	Chemler et al., 2007
(2S)-5,7-dihydroxy-4′-dihydroflavonol		p-Fluorocinnamic acid	0.75	Chemler et al., 2007
(2S)-5,7-dihydroxy-2′-fluoro-dihydroflavonol		o-Fluorocinnamic acid	4.5	Chemler et al., 2007
(2S)-4′-amino-5,7-dihydroxy-dihydroflavonol		p-Aminocinnamic acid	10.44	Chemler et al., 2007
**ISOFLAVONES**				
3′,4′,7-Trihydroxy isoflavone	*E. coli*	Daidzein	n.e.	Roh, 2013
**FLAVONES**				
2-(4-fluorophenyl)-5,7-dihydroxy-4H-chromen-4-onebenzopyran-4-one	*E. coli*	(2E)-3-(4-fluorophenyl)prop-2-enoic acid	30	Katsuyama et al., 2007a
2-(furan-3-yl)-5,7-dihydroxy-4H-chromen-4-one		(2E)-3-(furan-3-yl)prop-2-enoic acid hydrate	26	Katsuyama et al., 2007a
5,7-dihydroxy-2-(thiophen-3-yl)-4H-chromen-4-one		(2E)-3-(thiophen-3-yl)prop-2-enoic acid hydrate	46	Katsuyama et al., 2007a

*For structure details and metabolic steps see Figure 4*.

The construction of bacterial strains for the synthesis of unnatural flavonoid glycosides possessing interesting properties is possible through rationally designed approaches. This strategy involves the design of new molecules with a specific functionality, based on the ability to predict how the molecule's structure will affect its behavior through physical models. An interesting case was the use of a combination of glycosyl transferases to attach more than one glycosyl groups in the hydroxyl groups of the flavonoid backbone (diglycosides). In accordance with this idea, specific UGTs were expressed in *E. coli* for the production of 67 mg/L of quercetin 3-O-glucoside-7-O-rhamnoside and 67.4 mg/L of quercetin 3,7-O-bisrhamnoside (Kim et al., 2013a) (Figure 4, Table 2). The selection of a nucleotide specific glycosyl-transferase (UGT) and its expression into *E. coli* cells made the biosynthesis of the unnatural glycosylated quercetin analog, quercetin 3-O-6-deoxytalose (Yoon et al., 2012) possible. Moreover, an *E. coli* strain was engineered to produce 7-O-xylosyl naringenin when fed with naringenin (Simkhada et al., 2009). The strain was constructed through the overexpression of genes required for the biosynthesis of UDP-xylose as well as the 7-O-glycosyltransferase gene. Following a similar approach, Kim et al. (2012a) managed to produce 380 mg/L of quercetin 3-O-N-acetylglucosamine when the engineered *E. coli* strain was fed with quercetin. Methyl transferases have also been used for the production of methylated forms of flavonoids. The co-cultivation of a strain carrying an FLS gene and a strain carrying a 3-OMT successfully transformed the supplemented dihydrokaempferol or dihydroquercetin into kaempferol and quercetin respectively (Kim et al., 2010). Novel methyl-transferases for the methylation of an extra position of the flavonol backbone have also been engineered; 3,7-O-dimethylquercetin and 3,7-O-dimethylkaempferol were produced in *E. coli* at concentrations of 19.16 and 22 mg/L (Joe et al., 2010).

## Dynamic pathway regulation and metabolic control

Traditional metabolic engineering is largely focused on the over-expression of rate-limiting steps (Tai and Stephanopoulos, 2013), deletion of competing pathways (Stephanopoulos, 2012), managing ATP (Singh et al., 2011; Lan and Liao, 2012) and balancing redox and precursor metabolites (Singh et al., 2011). While these approaches have been proven effective in improving cellular productivity and yield, the engineered strains are often incapable of dynamically controlling gene expression and are susceptible to environmental perturbations. As heterologous pathways become larger and more complicated, it becomes increasingly difficult to optimize them with static regulatory control (Holtz and Keasling, 2010). Generally, optimization through static control is only applicable in a particular environment and any perturbations that are away from the prescribed condition would likely result in phenotype instability or suboptimal productivity.

Contrary to static control, native biological systems typically utilize dynamic regulatory networks to control metabolic flux in response to changing environments (Xu et al., 2013a). For example, one of the coherent strategies that occur in most biological systems is gene expression regulation through a negative/positive feedback loop (Afroz and Beisel, 2013). Mediated by a transcriptional regulator, a metabolic intermediate would act as a signaling molecule which would induce or repress the expression of enzymes responsible for its synthesis or consumption. Nature has developed this strategy in order to allow metabolic pathways to be dynamically controlled so that cellular resources can be efficiently utilized, regardless of the changing environment. In practice, this strategy could be applied to pathway optimization when accumulated toxic intermediates are detrimental to cell growth. Successful application of this optimization strategy would require the construction of hybrid promoters that are transcriptionally responsive to small intermediate molecules (Xu et al., 2014b). For example, Liao et al have designed and applied a regulatory circuit that could sense the glycolytic pathway hallmark metabolite acetyl-phosphate to control the lycopene biosynthetic pathway (Farmer and Liao, 2000) and generate oscillatory gene expression (Fung et al., 2005) as well as achieve artificial cell-cell communication (Bulter et al., 2004). Dhal et al. have employed stress-response promoters to improve farnesyl pyrophosphate production (Dahl et al., 2013) and Zhang et al. have constructed a fatty acyl-CoA responsive promoter to dynamically control gene expression involved in biodiesel synthesis (Zhang et al., 2012) in *E. coli*.

**Figure 5:**
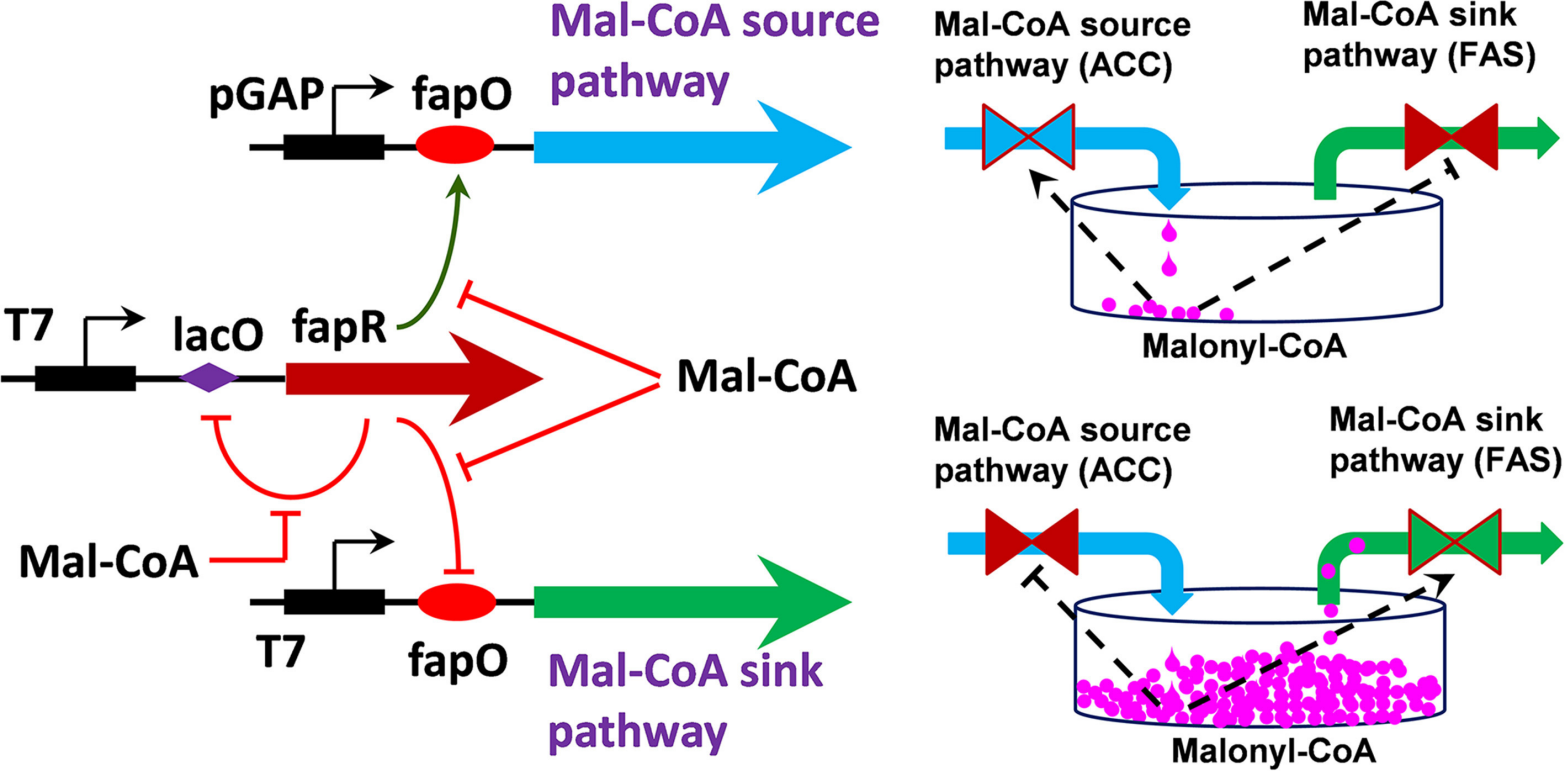
**Schematic representation of synthetic malonyl-CoA controller for dynamic tuning of metabolic flux in *E. coli* (Xu et al., 2014a)**. *FapR* activates gene expression from p*GAP* promoter and represses gene expression from T7 promoter at low level of malonyl-CoA. Binding of FapR with malonyl-CoA will switch gene source pathway (ACC) to malonyl-CoA sink pathway (FAS). *FapR*: malonyl-CoA responsive *Bacillus subtilis* fatty acids pathway transcriptional regulator; Mal-CoA, malonyl-CoA; FAS, fatty acids synthase; ACC, acetyl-CoA carboxylase; pGAP, *E. coli* GAP promoter; T7, bacteriophage T7 promoter; fapO, putative FapR binding site; lacO, putative lacI repressor binding site. Point arrows indicate activation and blunt-end arrows indicate repression.

Recently, Xu et al. (2014a,b) have reported a genetically-encoded metabolic switch that enables dynamic regulation of both the malonyl-CoA source pathway and the malonyl-CoA sink pathway (Figure 5). Engineering hybrid promoter-transcriptional regulator interactions led to the construction of two malonyl-CoA sensors that exhibit opposing transcriptional activities. Proper balancing of the transcriptional activity of the malonyl-CoA-up regulating promoter and malonyl-CoA-down regulating promoter resulted in an integrated malonyl-CoA switch rendering a bi-stable gene expression pattern. When this synthetic malonyl-CoA switch was implemented to control fatty acids production, the engineered strain could better balance the tradeoff between cell growth and product formation and demonstrated superior FA production profile. The control scheme in this study is the first report to use both the ON and OFF function to regulate cell metabolism. Unlike the report by Zhang et al. (2012) and Xu et al. (2014a,b) only used an “ON” function to control the expression of the fatty acid ethyl ester (FAEE) downstream pathway (fatty acyl-CoA and ethanol synthesis), where the FAEE upstream pathway (fatty acids synthesis) was left uncontrolled. Since malonyl-CoA is an essential precursor involved in the biosynthesis of flavonoids, polyketides and fatty acids-based biofuels (Xu et al., 2011, 2013b; Felnagle et al., 2012), the synthetic malonyl-CoA switch should facilitate the construction of strains for high yield production of malonyl-CoA-derived compounds.

## Metabolomics and NGS assisted bioinformatics for pathway mining

The vicious interaction between evolution and nature, equipped all organisms with appropriate genetic machinery to biosynthesize accessories of secondary metabolism, in order to withstand the pitfalls of their life cycle. Although part of this machinery has been identified, various parts remain uncovered. As analyzed earlier, all these secondary metabolites evolved from interconnected biosynthetic paths and followed various tailoring, condensing and decorating enzymatic interventions, which are responsible for their eventual categorization into phenylpropanoids, flavonoids, alkaloids, terpenoids, etc. In most of these biosynthetic pathways, core enzymes exist to put key biochemical reactions through for the production of the final product. The core enzymes of the flavonoid biosynthetic pathways are responsible for the production of the basal flavonoid skeleton. However, there is a plethora of other tailoring enzymes for the decoration of the core skeleton with extra chemical patterns differing in oxidation, acylation, methylation, or glycosylation patterns. Those are achieved by utilizing the oxido-reductases and acyl- or glycosyl-transferases available creating the different flavonoid classes—as described above, consisting of the overabundance of flavonoid compounds.

Emerging technologies in the fields of Metabolomics and Genomics offer massive assistance in the quest for new enzyme activities or even new metabolic pathways. On the one hand, improved mass spectrometry apparatuses let users identify chemical entities, and even extrapolate native intracellular concentrations. Such techniques coupled with processes for monitoring of metabolic transformations have allowed metabolomics to thrive within the context of enzyme function or pathway discovery in model or non-model organisms (Prosser et al., 2014).

On the other hand, the emergence of Next-Generation Sequencing technologies (NGS) has revolutionized functional genomics. Entire microbial genomes can now be sequenced, facilitating the discovery of biosynthetic gene clusters. The conserved features of the flavonoid core and decorating enzymes, that interact in such a manner to create flavonoid metabolons (Winkel, 2004; Crosby et al., 2011), may be used as queries in computer programs that compare primary biological sequence information to find matches (Altschul et al., 1990). This will help us to mine homolog flavonoid pathways in other species or individuals in general. The rapidly evolving NGS technologies (Hui, 2014) result in the loading of public databases with enormous amounts of organismal sequence data surpassing manual annotations. Having gigabases of data in their hands combined with metabolic profiling, functional genomics and system biology approaches, scientists can routinely examine *in-silico* and obtain in-depth knowledge in their attempt to further understand the flavonoid biosynthetic pathways. This may increase the potential of bio-engineering through identification of putative genes coding for enzymes with improved or novel activities involved in the flavonoid pathways. Along this line of reasoning, we should also note that deep transcriptome sequencing and metabolic profiling have allowed the identification of gene variants for anthocyanin biosynthesis in *Camellia chekiangoleosa* (Wang et al., 2014) and flavonoids and terpenoids biosynthesis in *Isatis indigotica* (Chen et al., 2013). Whenever lack of genomic information or developed genetic tools presents itself, the exploration of the molecular mechanism of flavonoid formation is faced with difficulties. Transcriptome sequencing can be an efficient approach to obtaining functional genomic information, which could contribute to pathway mining. Furthermore, generated NGS data will serve as information platforms for gene expression, genomics, and functional genomics. Efficient data analysis pipelines are required for all these applications in order for them to be established as routine activities, and more studies are needed aiming to address the robustness of these techniques (Morozova and Marra, 2008). Automated annotation needs to be as accurate as possible to avoid systematic errors generated by misjudging gene callings (Bakke et al., 2009). In other words, although sequencing has become easy, genome annotation has become more challenging: (a) NGS technologies yield short read lengths resulting in loss of contiguity, (b) sequencing of “exotic” genomes without pre-existing user-centric annotation data renders predictive gene-calling methods hard to train, optimize and configure (Yandell and Ence, 2012). Although genome annotation is not a point-and-click procedure, “do-it-yourself” projects are feasible using present-day tools. This has also allowed the discovery of a new range of enzymatic diversities, entirely new sequence classes and novel functionalities (Pollier et al., 2011).

## Concluding remarks

Flavonoids constitute a diverse group of secondary plant metabolites with fascinating compounds that display extraordinary antioxidant activity. Many of these compounds are used as pharmaceutical drugs or as nutraceutical supplements. Flavonoid engineering has greatly progressed over the past few years. Technical advances in gene discovery, functional genomics and the large-scale implementation of different combinatorial biosynthesis techniques in plants all promise a bright green future for the discovery and exploitation of plant derived products. Moreover, algorithmic tools, such as the software package OptForce, are already available to help researchers perform targeted genetic interventions in order to increase carbon flux through malonyl-CoA (Bhan et al., 2013). This is indeed the limiting precursor step for the overexpression of an array of heterologous pathways such as flavanones and polyketides in bacteria. Koffas' group has developed a sensor that manipulates two cellular pathways that regulate production of malonyl-CoA (Xu et al., 2014a) in real time. By utilizing the sensor-based dynamic regulation technique at the cellular level, researchers were able to maximize production of malonyl-CoA while minimizing any damage to the cell. This technique has been applied to fatty acid biosynthesis and seems very promising for flavonoids, since, by improving malonyl-CoA availability in *E. coli*, natural plant product titers can be improved as well. An alternative or a parallel approach would be to identify and exploit microorganisms that overproduce phenylalanine or tyrosine to minimize the input of carbon source during the fermentation procedure.

### Conflict of interest statement

The authors declare that the research was conducted in the absence of any commercial or financial relationships that could be construed as a potential conflict of interest.
